# Prevalence of Milk Fraud in the Chinese Market and its Relationship with Fraud Vulnerabilities in the Chain

**DOI:** 10.3390/foods9060709

**Published:** 2020-06-01

**Authors:** Yuzheng Yang, Liebing Zhang, Kasper A. Hettinga, Sara W. Erasmus, Saskia M. van Ruth

**Affiliations:** 1 Food Quality and Design Group, Wageningen University and Research, P.O. Box 17, 6700 AA Wageningen, The Netherlands; yuzheng.yang@wur.nl (Y.Y.); Kasper.Hettinga@wur.nl (K.A.H.); sara.erasmus@wur.nl (S.W.E.); 2 Wageningen Food Safety Research, part of Wageningen University and Research, P.O. Box 230, 6700 AE Wageningen, The Netherlands; 3 College of Food Science and Nutritional Engineering, China Agricultural University, P.O. Box 291, Beijing 100083, China; liebzhang@gmail.com

**Keywords:** China, Fourier transform-infrared spectroscopy, fraud vulnerability, milk adulteration, milk composition, one-class classifications

## Abstract

This study aimed to assess the prevalence of ultra-high-temperature (UHT) processed milk samples suspected of being adulterated on the Chinese market and, subsequently, relate their geographical origin to the earlier determined fraud vulnerability. A total of 52 UHT milk samples purchased from the Chinese market were measured to detect possible anomalies. The milk compositional features were determined by standardized Fourier transform-infrared spectroscopy, and the detection limits for common milk adulterations were investigated. The results showed that twelve of the analysed milk samples (23%) were suspected of having quality or fraud-related issues, while one sample of these was highly suspected of being adulterated (diluted with water). Proportionally, more suspected samples were determined among milks produced in the Central-Northern and Eastern areas of China than in those from the North-Western and North-Eastern areas, while those from the South were in between. Combining the earlier collected results on fraud vulnerability in the Chinese milk chains, it appears that increased fraud prevalence relates to poorer business relationships and lack of adequate managerial controls. Since very few opportunities and motivations differ consistently across high and low-prevalence areas, primarily the improvement of control measures can help to mitigate food fraud in the Chinese milk supply chains.

## 1. Introduction

The dairy industry in China has developed in parallel with the country’s economic growth. The average annual milk consumption of Chinese consumers reached 36 kg per capita in 2016, though this is still lower than the average world consumption [1]. Liquid milk is the main type of dairy product in the Chinese market, with more than 90% of the raw milk being processed to liquid milk products [2]. Moreover, the most popular liquid milk product, ultra-high-temperature (UHT) processed milk, accounted for more than half of the liquid milk consumption in 2018 [3].

The melamine infant formula incident in 2008 highlighted the vulnerability of the dairy industry in China and was a grave shock for this industry [4]. This incident resulted in great financial, as well as consumer confidence, losses for the sector. Milk, as a source of protein and calcium, plays an important role in the diet of Chinese consumers. Unfortunately, milk is one of the most commonly adulterated foods due to its popularity, production and sales in mass volume levels and the price paid for the product based on its composition [5]. Moreover, the number associated with the adulteration of dairy products made it the top-ranked product among the animal food products that are typically subjected to fraud [6]. The rapidly growing economy in China has led to a gradual shift of concerns about food security to food safety [7]. Linked to food safety is also the prevalence of food fraud, as the adulteration of milk products, depending on the type of adulterant used, can have safety implications for a food product.

Food fraud has become a widely acknowledged concern, not only within the food supply chain, but also more widespread, for instance, by consumers. Recent studies on Chinese consumers’ preferences showed that Chinese consumers are concerned about the risk of domestic milk products and, especially, infant milk formula [8] and therefore prefer to buy “foreign milk powder” instead of domestic products [9]. Melamine is not the only milk adulterant. Milk adulterations range from very simple, such as dilution, to very complex, such as synthesizing milk with urea, vegetable oil, detergents and other chemical compounds [10]. A number of substances have been listed as potential milk adulterants by different scholars [10,11].

In our previous studies, technical opportunities were identified as medium-high risk factors in the investigations of the fraud vulnerability of milk supply chains, whereas the detectability of milk adulteration was a main concern of the participants in the supply chain [12,13]. Various advanced techniques have been developed for milk authentication. For example, chromatographic methods combined with mass spectrometry (MS) have been used for detecting milk adulteration with nitrogen-rich compounds [14,15] and vegetable oils [16], digital imaging for milk protein determinations [17] and the detection of hydrogen peroxide in milk [18], proton transfer reaction mass spectroscopy (PTR-MS) and stable isotope ratio mass spectroscopy (IRMS) for the discrimination of organic milk [19,20] and nuclear magnetic resonance (NMR) for the nontargeted detection of multiple adulterants in milk powder [21]. Moreover, infrared spectroscopy-based techniques have become the most commonly used method for determining food authenticity, and they are considered as alternatives to reference methods [22]. Furthermore, automated equipment based on Fourier transform-infrared (FTIR) spectroscopy has been developed to determine milk composition, providing high analytical capacity and low operational costs [23]. FTIR spectroscopy is extensively used worldwide for milk quality control, because little sample preparation is needed, and the analysis is rapid. In combination with statistical analysis, FTIR spectroscopy has been applied to identify several milk adulterants, such as melamine [24], whey protein [25], sodium bicarbonate, sodium citrate and corn starch [26].

Considering the previous milk fraud incidents in China and the uprising public concerns from the Chinese consumers, there is an urgent demand for information on the integrity (i.e., safety, quality and authenticity) of milk products on the Chinese market to restore consumers’ trust. As previous studies have already addressed food safety issues [27,28,29,30], the current study aims to evaluate the occurrence of milk adulteration in China and relate the prevalence of the milk fraud to fraud vulnerability profiles. The occurrence of milk adulteration was evaluated by anomaly detection. Anomaly detection was firstly conducted based on individual measurement parameters that are commonly used in practice, using both univariate and multivariate approaches. For both approaches, the data of a control group of genuine UHT milk samples was used in combination with groups of protein-rich, nitrogen-based and carbohydrate-based milk adulterants, as well as nonallowed preservatives at various concentration levels to set boundaries. Finally, commercial UHT milk samples from different regions in China were tested against the boundaries using both developed approaches. Fraud prevalence was related to the geographical origin of the samples and compared with previously established fraud vulnerabilities for the geographical areas.

## 2. Materials and Methods

### 2.1. Sample Collection

A total of twelve UHT milk samples from five different brands were purchased from local supermarkets in Beijing in November 2018. They were from the top 10 dairy-processing enterprises in China and considered as the reference samples. Among these twelve samples, four samples were labelled as premium quality (protein content > 3.5%) and produced in the North of China, four were of normal quality and also produced in the North and the remaining four samples were of normal quality and produced in the South. Accordingly, three milk pools were prepared: the first milk pool (Pool A) was a mixture of the four samples of premium quality, the second pool (Pool B) a mixture of the four samples of normal quality from the North of China and the third pool (Pool C) a mixture of the four samples of normal quality from the South of China. The ratio of the four milk samples in each pool was 1:1:1:1 *w*/*w*. Three milk pools of 100 g each were prepared, to which an adulterant was added at several concentrations. The twelve reference samples and the three milk pools were from major producers and, hence, considered as the control samples (i.e., nonadulterated). These 15 samples comprised the training set.

**Figure 1:**
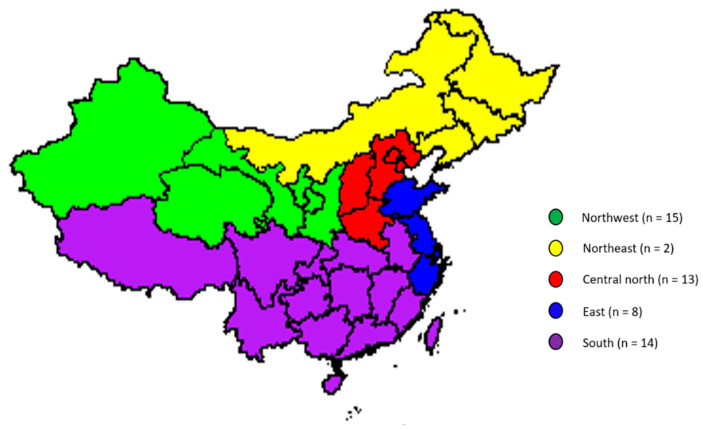
The geographical distribution of the market survey samples.

A total of 52 commercial UHT milk samples were purchased from local markets (in Beijing) and e-commerce (across China) during the winter of 2018/2019 (December 2018 to January 2019), and these samples comprised the market survey test set. The distribution of the geographical origin of the market survey samples is shown in Figure 1.

### 2.2. Adulterations and Measurements

Following the same procedure of a comparable study that was conducted on milk samples from the Dutch market, the same adulterants and the same adulteration levels were applied [31]. A total of 24 adulterants were used, which were categorized into five groups as follows: (1) protein-rich adulterants including whole milk powder (WMP), skimmed milk powder (SMP), whey protein isolate (WPI), pea protein isolate (PEA) and soy protein isolate (SOY); (2) nitrogen-based adulterants including urea (URE), melamine (MLM), ammonium sulphate (AS), ammonium chloride (AC) and dicyandiamide (DIC); (3) carbohydrate-based adulterants including sucrose (SU), glucose (GLU), corn starch (ST), lactose (LAC), fructose (FRU), maltodextrin (MD) and arrowroot powder (AR); (4) preservatives including sodium citrate (CIT), sodium carbonate (CAR), sodium bicarbonate (BIC), sodium hydroxide (HYD), formaldehyde (FMD) and hydrogen peroxide (PX) and (5) water. Both single and combined adulterations were conducted. The single adulterations were carried out at four levels for each adulterant. The formulas and detailed plan for the single adulterations are provided in Table S1 (Supplementary Materials). The combined adulterations were conducted in two steps: first, 40 g water was added to 100 g of a milk pool sample; then, one of the adulterants from either the protein-rich, nitrogen-based or carbohydrate-based adulterant category was added to the diluted milk pool to increase the apparent protein content with 40% *w*/*w* (adulterant protein/milk protein content for the protein-rich and nitrogen-based adulterations) or to increase the apparent total solids content with 40% *w*/*w* (adulterant total solids (TS)/milk TS content for the carbohydrate-based adulterations). The detailed information of the combined adulteration is provided in Table S2 (Supplementary Materials). Ultimately, a total of 288 single-adulterated samples and 51 combined-adulterated samples were prepared. These 339 adulterated samples were considered as the adulterant test set.

MilkoScan FT120 equipment (Foss Electric, Hilleroed, Denmark) was used to measure the milk composition. The equipment is based on the FTIR technique and reports a series of milk compositional parameters, namely protein, fat, lactose, total solids (TS), solids nonfat (SNF) content, density and freezing point depression (FPD). The FTIR spectra were not acquired separately because of the limitation of the instrument used. All the samples were prepared at room temperature and measured within two hours after preparation.

### 2.3. Statistical Analysis

#### 2.3.1. Univariate Analysis: Determination of Boundaries for Each Variable

The mean and standard deviation (SD) for the seven variables (i.e., protein, fat, lactose, total solids, solids nonfat, freezing point depression and density) were calculated based on the 15 control samples. Next to that, the values of the 0.5th and 99.5th percentiles for each variable were used to set boundaries for anomaly detection.

According to a programme of measurements of over 3 million raw milk samples for legislatorial control (Zuivelverordening, 2000), the standard deviation of this large sample set for the seven variables is roughly double the values of the control samples in this study. To adapt the variance of the control samples in a practical way, the data of the 15 control samples was transformed into a variance-adjusted dataset, where the mean value for each variable remained the same, but the SD value was adjusted to twice the measured SD. The new dataset was converted from the measured data for each variable separately using Equation (1):

(1) $$X_{new}=\left(\frac{X-\mu }{\sigma }\times 2\sigma \right)+\mu$$

where $X_{new}$ is the variance-adjusted data, *X* is the measured data for the control samples, μ is the mean value of the 15 control samples and σ is the SD of the 15 control samples. Next, the variance-adjusted boundaries were determined using the values of 0.5th percentile and 99.5th percentile for the seven variance-adjusted variables.

The results of the measured and variance-adjusted datasets are shown in Tables S3 and S4 (Supplementary Materials), respectively. Both the measured boundaries and variance-adjusted boundaries were then utilized for both the adulterant test set and market survey test set. The univariate calculations were performed using Microsoft Excel 2016 (Microsoft, Redmond, WA, US).

#### 2.3.2. Multivariate Analysis: Determination of Boundaries for Milk with One-Class Classification Models

One-class classification (OCC), which focuses on a single target class, has become a common modelling approach for the verification of food authenticity [32]. Three one-class classification (OCC) models were applied in this study, namely k-nearest neighbours (KNN), soft independent modelling of class analogies (SIMCA) and support vector machine (SVM). KNN has no requirement for the data distribution and is robust to noisy training data, and hence, it is suitable for analysing small training sets [33]. SIMCA focuses more on the similarities among samples within a class and is thus widely used for OCC models [34]. SVM is another fitting approach that can be applied to datasets with a limited number of training samples [35]. SVM evaluates the distance from an object to the boundary. For this study, the Gaussian radial basis function (RBF) kernel was used to determine the boundary for the SVM model.

The measured dataset (*n* = 15) and variance-adjusted dataset (*n* = 15) of the control samples were separately used as the training set for the model development. The training set was subjected to leave-30%-out cross-validation with 100 repetitions. The dataset was preprocessed by means of autoscaling. Next to that, the three classifiers (KNN, SIMCA and SVM) were applied. A significant level of 1% (*p* < 0.01) was used for determining the critical classification thresholds. The adulterant test set, comprising 339 adulterated milk samples, was then subjected to the developed models. The three OCC models were evaluated applying the following parameters: the k value for the KNN model was selected from consecutive numbers 1–10; the number of factors *n* for SIMCA was selected from consecutive numbers 1-7; γ in the Gaussian radial basis function (RBF) kernel for the SVM was selected from 10^−9^, 10^−8^, 10^−7^, 10^−6^, 10^−5^, 10^−4^, 10^−3^, 10^−2^, 10^−1^ and 1. The average value of the percentages of correctly assigned samples for the cross-validation set and adulterant test set was used to evaluate the overall performance of the models. The optimal parameter for the best performing model was determined accordingly. Next, the market survey test set, comprising 52 samples, was subjected to the selected models. The OCC model development in this study was performed using R 3.6.1 (R Foundation for Statistical Computing, Vienna, Austria).

#### 2.3.3. Exploratory Analysis and Regression Model

The result of the compositional features was subjected to principal component analysis (PCA) to visualize the grouping of the control samples and market survey samples after preprocessing by autoscaling. The PCA was performed by R 3.6.1 (R Foundation for Statistical Computing, Vienna, Austria). Principal component regression (PCR) with leave-one-out cross-validation was conducted between the geographical prevalence of the suspected samples and the result of the food fraud vulnerability assessment in the corresponding areas. The mean ranks of the scores of fraud factors for four of the main milk production areas in China (i.e., Central-North, Northeast, Northwest and East of China) from a previous fraud vulnerability assessment study [13] were used to develop a model to predict the percentage of suspected samples in these areas after preprocessing by autoscaling. The PCR was performed using Pirouette 4.5 (Infometrix Inc., Bothell, WA, USA).

## 3. Results and Discussion

### 3.1. Control Samples

#### 3.1.1. Natural Variation of the Control Samples

A certain degree of variation in the milk composition was observed among the three milk pools analysed by FTIR spectroscopy (Table 1). All the measured compositional features of the premium milk pool (pool A) were higher than those of the normal milk pools from both the North and the South (pool B and C). Generally, the fat and lactose contents of the control milk are in agreement with those of the raw milk in China, which are 3.6% to 4.2% *w*/*w* and 4.7% to 5.1% *w*/*w*, respectively [36,37]. However, the protein content of the control samples (3.4% to 3.7% *w*/*w*) was higher than that of the raw milk produced by Chinese Holstein cattle, which is approximately 2.9% to 3.3% *w*/*w* [1,36]. This difference may have been caused by protein standardization techniques used during the processing of the milk—for instance, flash evaporation [38]. It may also be due to the use of raw milk of a higher protein content from other dairy cattle breeds. A difference in milk composition was observed between the commercial UHT milk samples from the Dutch and Chinese markets. The means of the fat content and lactose content of the Chinese samples (4.0% and 5.1% *w*/*w*, respectively) were slightly higher than those of the Dutch ones (3.8% and 4.7% *w*/*w*, respectively), while the mean values for the protein content of the milk from the two countries were more or less the same (3.5%–3.6% *w*/*w*) [31].

#### 3.1.2. Control Samples and Univariate Detection Approach

The univariate boundaries based on the measured dataset and variance-adjusted dataset are presented in Table 1. The 15 control samples were tested by the two sets of boundaries. According to the measured dataset, five compositional values of the control samples out of 105 measurements exceeded the measured boundaries, including four that exceeded the upper boundaries and one that exceeded the lower boundary. The samples exceeding the upper boundaries concerned protein, fat, SNF and the lactose content (control samples 2–4), while control sample 8 exceeded the lower boundary of the TS content (Table S3, Supplementary Materials). It seems that the samples of which the compositional features exceeded the upper boundaries are due to features generally occurring in premium quality milk; in other words, they are in the top 0.5% of the distribution of the control samples with regard to the protein, fat, SNF and/or lactose concentration. Considering the lactose content of raw milk is quite stable, ranging from 4.5% to 5.0% *w*/*w* [39,40], and would not be intentionally adjusted during the milk-processing, it is believed that the high lactose content of the premium milk product was caused by the use of flash evaporation, which is sometimes used for the production of premium milk in China [38]. Such flash evaporation would also lead to an increase of the protein, fat and SNF contents by water removal, as was found for samples 2–4. As samples 2–4 were thus exceeding the boundaries for reasons other than adulterations, they were kept in the control group. Considering the large variance that would be found in practice among unadulterated samples, the variance-adjusted dataset was also applied, against which all the 15 controls samples were considered normal.

#### 3.1.3. Control Samples and the Multivariate Detection Approach

One-class classification models using three classifiers (KNN, SIMCA and SVM) were calculated, and for each classifier, the model with the best performance was selected. The results of the selected models are shown in Table 2. For all the three classifiers in both scenarios (i.e., the measured dataset and variance-adjusted dataset), the samples of the training set were 100% correctly classified. The KNN classifier performed a bit better for the cross-validation set, achieving 92% and 93% accuracy for the model of the measured dataset and variance-adjusted dataset, respectively. Combining the performance of the models in both scenarios, KNN was selected as the best classifier for the OCC model for further analysis.

To summarise, based on the variation of the composition of both the control samples in this study (i.e., the measured dataset) and a more practicable scenario (i.e., the variance-adjusted dataset), the univariate boundaries and multivariate models were determined, respectively. The same models were then subjected to the adulterant test set and market survey set.

### 3.2. Adulterants

#### 3.2.1. Adulterants and the Univariate Detection Approach

**Figure 2:**
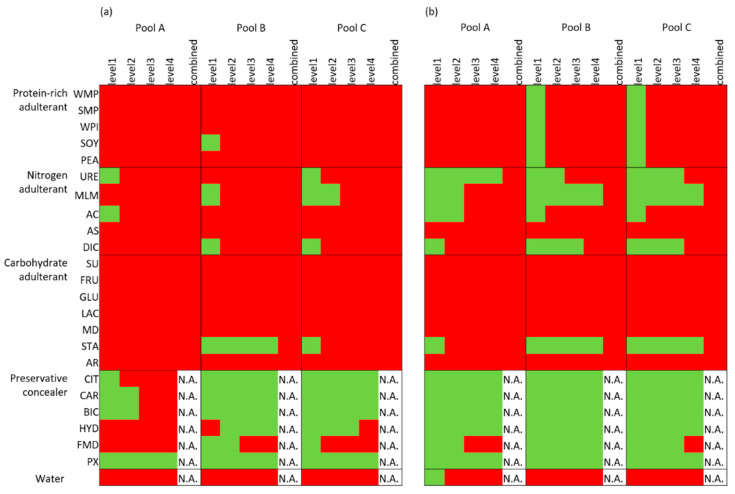
The results of the adulterant test sets for the three milk pools based on (**a**) the measured boundaries of the univariate detection and (**b**) the variance-adjusted boundaries of the univariate detection, indicating the potential to identify suspected milk adulterations. The samples with all results within the boundaries are coloured green, while the rest is coloured red. The full names of the adulterants are shown in the abbreviations list. For interpretation of the different colours, refer to the web version of the paper. N.A., not applicable.

To test the detection capacity of the developed approaches, the univariate boundaries were first applied to the adulterant test set. As expected, the univariate boundaries of the measured dataset flagged more adulterations than the variance-adjusted boundaries, as shown in Figure 2. Both boundaries flagged the high concentrations (levels 2–4) of the protein-rich adulterations and almost all carbohydrate adulterations (except for starch adulteration). Furthermore, the measured boundaries flagged high concentrations (levels 3–4) of the nitrogen adulterants, while the variance-adjusted boundaries had a lower performance for these adulterations. The water dilutions were almost universally flagged by both boundaries. Most of the preservative concealers passed unnoticed for both types of boundaries (Figure 2).

#### 3.2.2. Adulterants and Multivariate Detection Approach

**Figure 3:**
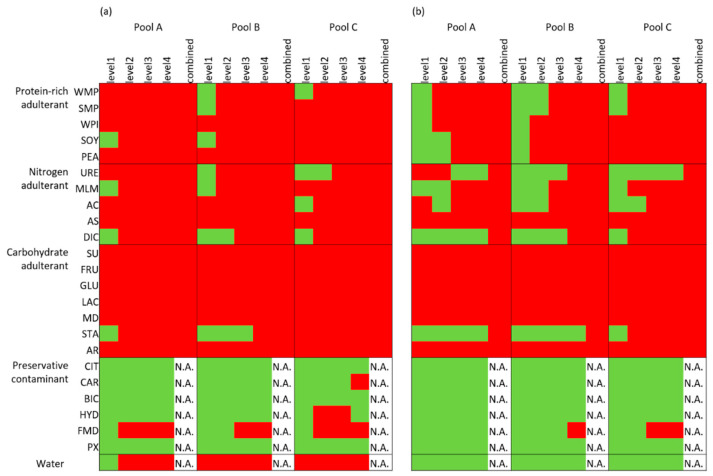
The results of the adulterant test sets for the three milk pools based on the threshold of the of k-nearest neighbour (KNN) model developed from (**a**) the measured dataset and (**b**) the variance-adjusted dataset, indicating the classification of milk adulterations. The samples with results within the threshold are coloured green, while the rest are coloured red. The full names of the adulterants are shown in the abbreviations list. For interpretation of the different colours, refer to the web version of the paper.

The selected KNN models were also applied to the adulterant test set. Similar to the scenario of the univariate detection, the KNN model based on the measured dataset also raised more flags than that based on the variance-adjusted dataset for the various adulterations, as shown in Figure 3. All carbohydrates, except for the starch adulterations, were flagged by both models. When considering the performance of the KNN model of the measured dataset, the average specificities of the model of the 100 repetitions for the protein-rich adulterations (95%) and carbohydrate adulterations (93%) were slightly higher than that of the nitrogen adulterations (86%)—only the ammonia sulphate adulterations in the latter group were fully flagged. The KNN model of the measured dataset flagged most water dilutions, while that of the variance-adjusted dataset flagged no water dilutions at all. The specificities of both models for the preservative adulterations were very low.

#### 3.2.3. Comparison of Approaches

For both the univariate and multivariate approaches, the ones based on the measured dataset flagged more adulterated samples than those based on the variance-adjusted dataset, due to the measured dataset having less variance. All the developed criteria succeeded to flag carbohydrates, except for the starch adulterations, and also flagged protein-rich adulterations at the higher levels. Both approaches based on the measured dataset could identify nitrogen adulterations at higher levels as well. In addition, it is noted that the multivariate approach did not perform better than the univariate approach in distinguishing the milk adulterations.

### 3.3. Market Survey Samples: What Type of Suspected Milk Samples are Discovered Using the Developed Approaches?

#### 3.3.1. Suspected Samples Flagged by the Univariate Detection Approach

As the developed approaches showed different abilities in detecting adulterations, they were all applied to the market survey samples. Out of the 52 samples from the market survey, 37 samples were flagged according to the univariate boundary of the measured dataset (Table 3). Their compositional results showed that the protein, fat and lactose contents were the main parameters that exceeded the boundaries. Only two samples were flagged for exceeding the upper boundary based on the lactose content (samples 32 and 37). As discussed in Section 3.1.2, the lactose content of raw milk is rather stable and will be below 5.0% *w*/*w*. The extremely high lactose content of these two suspected samples (>5.4% *w*/*w*) was likely caused by some kind of manipulation during processing, by either legal ways like flash evaporation or illegal ways like carbohydrate or dairy powder additions. The other 35 suspected samples were flagged, because their compositional variables exceeded the lower boundaries. Among these suspected samples, 32 samples were observed to be deficient in proteins, 15 samples deficient in fat and 15 samples deficient in lactose. It is not surprising that the TS and SNF contents of these suspected samples exceeded the lower boundary as well. These results indicate that the flagged samples were deviating from the control samples with respect to multiple compositional parameters, including protein, fat and/or lactose contents. However, it should be noted that the variance of the measured dataset is smaller than that faced in practice, which likely resulted in more genuine samples being misclassified. This would hence increase the workload of further checking these samples and lower the users’ acceptance. In addition, although the protein or fat contents of these samples were lower than the boundary, they were not exceeding the lower limit of the national food safety standard for sterilized milk, where 2.9% *w*/*w* is stipulated for the protein content and 3.1% *w*/*w* for the fat content [41]. It seems that the boundaries based on the measured dataset were thus too strict for practical use.

A total of twelve samples were flagged according to the univariate boundary based on the variance-adjusted dataset (Table 3). Eleven of these flagged samples were lower in protein contents, exceeding the lower boundary (3.13% *w*/*w*) of the variance-adjusted dataset. In addition, it was observed that some of the other compositional features such as the fat, total solids, density or FPD of these samples also exceeded the respective lower boundaries. There are probably multiple reasons for the low protein contents in these UHT milk products. One is that the raw milk used to produce the final products could have been low in protein contents. Considering there is no prohibition on adjusting the protein, fat, or lactose contents of UHT milk in China, another reason may be that the milk composition was changed during the processing—for instance, removing part of the milk fat would result in a lower fat content. It is also possible that the milk samples were diluted, although this would be a violation of the national food safety standard for sterilized milk, if the milk was diluted during the processing but labelled as “pure milk” on the package of the final product [41]. Since the milk samples in this study were all labelled “pure milk”, the lower protein and fat contents of the flagged milk samples might be indicators of potential milk manipulation. Therefore, these twelve samples were suspected of quality or fraud issues. In addition, the FPD of one suspected sample (sample 1) exceeded the boundary of the variance-adjusted dataset. The freezing point is principally affected by the lactose and dissolved salts in the milk and is very constant due to its effect on the osmotic pressure of milk. The addition of water would reduce the concentration of these compounds and lead to a change of the freezing point towards zero [42]. Taking the reasons above into consideration, sample 1 was most likely adulterated by water dilution.

#### 3.3.2. Suspected Samples Flagged by the Multivariate Detection Approach

**Figure 4:**
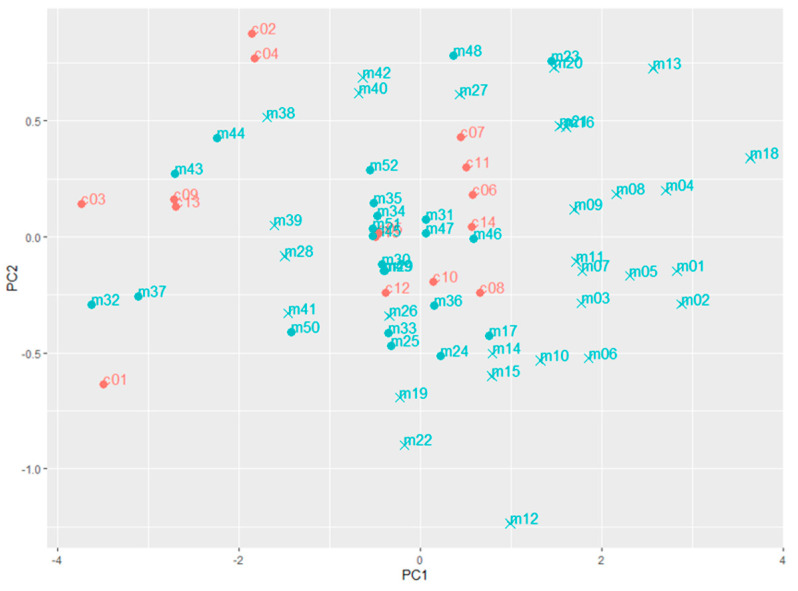
The principal component analysis (PCA) plot of the first two PC dimensions of the control samples (red points) and the market survey samples (blue points) based on the results obtained from the MilkoScan measurements. The suspected samples flagged by the k-nearest neighbours (KNN) model of the measured dataset are cross-shaped (x).

According to the KNN OCC model developed with the measured dataset, 29 market survey samples were flagged (Table 3). Among these suspected samples, the compositional features of 24 samples (samples 1–16, 18–22 and 26–28) were in-line with the univariate analysis, which exceeded the lower boundary based on the measured dataset. The five other samples (samples 38–42), of which the compositional features were within the univariate boundaries, could be explained by the characteristics of the KNN algorithm. In the classification phase of the KNN OCC model, for each object in the test set, the *k* (*k = 3* in this study) nearest training set vectors (the control samples) are determined, the distance between them calculated and the classification is then done by comparing the distance between the object and its *k* nearest neighbours to a predetermined threshold [43]. To visualise the variation among samples, a PCA was performed with the measured data of the control samples and market survey samples, as presented in Figure 4. Although the samples 38–42 were located in the middle of the control samples, their *k* nearest neighbours were not as close as the nonflagged samples, explaining why they were flagged by the KNN OCC model. It is noted that Figure 4 shows only the first two PCs of the PCA, instead of the “complete distribution”, as used for the KNN model; however, it does provide a visualisation of the variation among samples.

Three samples (samples 38, 39 and 43) were flagged by the KNN OCC method developed from the variance-adjusted dataset (Table 3). Similar to the scenario as described for the measured dataset, the spatial distance between these three flagged samples and their *k* (*k = 3* in this study) nearest neighbours is larger than the determined threshold of the models based on the variance-adjusted dataset. As a result, these samples were flagged as differing from the control group.

#### 3.3.3. Overall Suspected Samples of the Market Survey Set

A total of 43 samples of the market survey set (*n* = 52) were flagged by the developed approaches (Table 3). Some exceeded only the boundary of one feature, while in most cases, samples exceeded multiple boundaries. Since more variation, as would be expected in practice, has been considered with the application of the variance-adjusted dataset, more attention should be paid to the samples exceeding the variance-adjusted boundaries. As every approach has its limits, combining multiple criteria simultaneously to detect suspected samples would provide a new perspective. In the end, the samples that violated most criteria, i.e., three out of four, were considered as the suspected samples among the market survey samples, which resulted in twelve suspected samples (samples 1–2, 4–6, 12–15, 18, 19, 21 and 26). Among these samples, four were produced in the Central-Northern area of China, three in the Eastern area, two in the North-Western area and three in the Southern area.

### 3.4. Relation Between the Origin of the Suspected Milk and the Previously Determined Fraud Vulnerability

As shown in Table 4, the percentages of the suspected samples in the North-Eastern and North-Western areas (0% and 13%, respectively) were lower than those in the Central-Northern and Eastern areas (31% and 38%, respectively). This is in-line with a study on the regional distribution of reported food fraud incidents, where more food fraud scandals or incidents were reported in the provinces of the Central-Northern and Eastern areas compared to the North-Western and North-Eastern areas in China [6]. The fraud vulnerabilities of the Northern and Eastern areas have been identified in a previous study [13]. The fraud factors showing significant differences between the milk production areas are presented in Table 4, following the result of a PCR model that aimed to relate the determined fraud vulnerability to the number of suspected samples in certain areas.

#### 3.4.1. Relation Between the Origin of the Suspected Milk and the Fraud Opportunities and Motivations

As presented in Table 4, the milk chain actors from the East and Central-Northern areas, where more suspected UHT milk samples were flagged, stated before that they had poorer business relationships within the supply chain than those in other areas of China (fraud factor 4). Since a good relationship between the actors in the milk supply chain can positively affect information-sharing, this can help to keep the supply chain transparent [44], which may additionally play a role in reducing the risk of fraud in certain areas. Conversely, the situation may deteriorate. As a consequence, the regression vector of the PCR showed positive coefficients between the business relationship (fraud factor 4) and the percentage of the suspected samples, indicating that a high rank of the factor would contribute to the higher percentages of suspected samples in these areas.

#### 3.4.2. Relation Between the Origin of the Suspected Milk and the Counteracting Controls

The results of our study revealed that two managerial control measures, lack of an ethical code of conduct and lack of integrity screening of the employees, and one technical control measure (lack of fraud contingency plans) related to the higher prevalence of suspected samples from the milk production participants in the East and Central-Northern areas (Table 4). Well-established controls may mitigate against food fraud. However, if they are lacking in combinations with increased opportunities and motivations, companies become increasingly vulnerable to fraud [45].

## 4. Conclusions

The study demonstrated the occurrences of suspected adulterated UHT milk samples in various parts of China and their relationships with previously established fraud vulnerability of businesses operating in those areas. Twelve (out of 52) samples in a market survey were suspects with quality or fraud-related issues, of which one is highly suspected of being adulterated by a dilution with water. The relative prevalence of suspect samples was higher in milks produced in the Central-Northern and Eastern areas than in those produced in the North-Western and North-Eastern areas, while those of the Southern area were in between. The underlying factors contributing to this higher vulnerability are poorer business relationships and a lack of adequate managerial controls.

## Figures and Tables

**Table 1:** Means and standard deviation (SD) of the compositional features of the control samples measured by standardised Fourier transform-infrared spectroscopy and the boundaries based on the measured dataset and variance-adjusted dataset.

Dataset		Compositional Features ^a^						
		Protein (% *w*/*w*)	Fat (% *w*/*w*)	TS (% *w*/*w*)	SNF (% *w*/*w*)	Lactose (% *w*/*w*)	Density (g/L)	FPD (°C)
Pools	Pool A (premium, North)	3.69	4.05	13.72	9.73	5.30	1034	0.567
	Pool B (normal, North)	3.44	3.75	12.79	9.05	4.88	1031	0.524
	Pool C (normal, South)	3.47	3.83	13.04	9.24	5.04	1032	0.544
Measured dataset	Mean	3.54	3.95	13.24	9.33	5.06	1032	0.543
	SD	0.15	0.24	0.51	0.35	0.21	1	0.022
Measured boundary	Lower boundary	3.33	3.60	12.57	8.94	4.80	1031	0.516
	Upper boundary	3.73	4.42	14.03	9.85	5.39	1035	0.576
Variance-adjusted boundary	Lower boundary	3.13	3.26	11.90	8.55	4.54	1030	0.489
	Upper boundary	3.93	4.90	14.82	10.37	5.72	1038	0.608

^a^ TS, total solids; SNF, solids nonfat and FPD, freezing point depression.

**Table 2:** The results of the one-class classification models developed from the measured dataset and variance-adjusted dataset. All the present values refer to the average of 100 repetitions of cross-validation for the corresponding dataset.

Model	Performance for Dataset	Correctly Assigned Samples ^a^ (%)		
		KNN	SIMCA	SVM
Model developed from the measured dataset	Training set	100	100	100
	Cross-validation set	92	88	90
	Adulterant test set	77	75	79
	Overall performance	84	81	84
Model developed from the variance-adjusted dataset	Training set	100	100	100
	Cross-validation set	93	91	92
	Adulterant test set	66	60	63
	Overall performance	79	75	77

^a^ KNN stands for k-nearest neighbours; the KNN model with the best performance was estimated with *k* = 3 for both the measured and variance-adjusted datasets. SIMCA stands for soft independent modelling of class analogies; the SIMCA model with the best performance was estimated with the number of the factors *n* = 3 for both the measured and variance-adjusted datasets. SVM stands for support vector machine; the SVM model with the best performance was estimated with γ=0.1 for both the measured and variance-adjusted datasets.

**Table 3:** The results of the suspected samples in the market survey set according to the developed detections ^a^.

ID	Protein (% *w*/*w*)	Fat (% *w*/*w*)	TS (% *w*/*w*)	SNF (% *w*/*w*)	Lactose (% *w*/*w*)	Density (g/L)	FPD (°C)	Area	Province	Univariate Boundaries		Multivariate Models (KNN)	
										Measured Dataset	Variance-Adjusted Dataset	Measured Dataset	Variance-Adjusted Dataset
1	3.09 **	3.56 *	12.06 *	8.42 **	4.58 *	1029 **	0.488 **	N	Tianjin	7	4	yes	no
2	3.07 **	3.48 *	11.94 *	8.37 **	4.55 *	1029 **	0.490 *	NW	Xinjiang	7	3	yes	no
3	3.28 *	3.49 *	12.25 *	8.69 *	4.65 *	1030 *	0.515 *	NW	Xinjiang	7	0	yes	no
4	3.05 **	3.79	12.35 *	8.51 **	4.71 *	1029 **	0.498 *	N	Henan	6	3	yes	no
5	3.12 **	3.61	12.18 *	8.50 **	4.62 *	1030 *	0.492 *	N	Tianjin	6	2	yes	no
6	2.99 **	3.43 *	12.06 *	8.58 *	4.85	1030 *	0.512 *	E	Zhejiang	6	1	yes	no
7	3.14 *	3.70	12.32 *	8.56 *	4.67 *	1030 *	0.494 *	N	Henan	6	0	yes	no
8	3.28 *	3.74	12.49 *	8.68 *	4.64 *	1030 *	0.513 *	NW	Xinjiang	6	0	yes	no
9	3.17 *	3.87	12.54 *	8.62 *	4.69 *	1030 *	0.505 *	NW	Xinjiang	6	0	yes	no
10	3.22 *	3.40 *	12.19 *	8.72 *	4.74 *	1031	0.497 *	S	Chongqing	6	0	yes	no
11	3.33	3.60 *	12.39 *	8.73 *	4.63 *	1030 *	0.492 *	S	Yunnan	6	0	yes	no
12	2.95 * *	3.05 **	11.78 **	8.69 *	4.99	1031	0.510 *	E	Jiangsu	5	3	yes	no
13	3.14 *	4.16	12.75	8.55 *	4.65 *	1029 **	0.496 *	N	Tianjin	5	1	yes	no
14	3.03 **	3.57 *	12.34 *	8.74 *	4.96	1031	0.513 *	S	Yunnan	5	1	yes	no
15	3.17 *	3.40 *	12.29 *	8.85 *	4.93	1031	0.510 *	N	Shanxi	5	0	yes	no
16	3.15 *	4.15	12.81	8.62 *	4.70 *	1030 *	0.500 *	NW	Xinjiang	5	0	yes	no
17	3.27 *	3.53 *	12.41 *	8.83 *	4.80	1031	0.514 *	S	Yunnan	5	0	no	no
18	3.01 **	3.63	12.26 *	8.58 *	4.81	1028 **	0.538	S	Hubei	4	1	yes	no
19	3.12 **	3.51 *	12.47 *	8.95	5.07	1032	0.513 *	S	Yunnan	4	1	yes	no
20	3.25 *	4.21	13.05	8.81 *	4.80	1030 *	0.505 *	N	Hebei	4	0	yes	no
21	3.12 **	4.06	12.86	8.78 *	4.91	1030 *	0.523	NW	Shaanxi	3	1	yes	no
22	3.25 *	3.31 *	12.32 *	8.97	4.96	1032	0.520	N	Beijing	3	0	yes	no
23	3.37	4.16	13.09	8.89 *	4.75 *	1030 *	0.525	NW	Gansu	3	0	no	no
24	3.30 *	3.47 *	12.50 *	8.99	4.94	1032	0.523	S	Guangdong	3	0	no	no
25	3.26 *	3.58 *	12.67	9.07	5.06	1032	0.531	N	Hebei	2	0	no	no
26	3.09 **	3.75	12.76	9.00	5.16	1032	0.541	E	Shandong	1	1	yes	no
27	3.26 *	4.23	13.24	9.00	4.97	1031	0.570	NW	Qinghai	1	0	yes	no
28	3.32 *	3.97	13.24	9.29	5.21	1033	0.536	S	Yunnan	1	0	yes	no
29	3.29 *	3.84	12.91	9.05	5.00	1032	0.538	E	Shandong	1	0	no	no
30	3.29 *	3.83	12.94	9.06	5.05	1032	0.532	N	Beijing	1	0	no	no
31	3.30 *	3.91	12.95	9.03	4.97	1032	0.524	N	Hebei	1	0	no	no
32	3.63	3.88	13.67	9.83	5.42 †	1035	0.573	NE	Heilongjiang	1	0	no	no
33	3.34	3.57 *	12.73	9.13	5.03	1032	0.524	NW	Ningxia	1	0	no	no
34	3.26 *	3.99	13.10	9.11	5.08	1032	0.539	NW	Xinjiang	1	0	no	no
35	3.32 *	3.97	13.17	9.19	5.11	1032	0.538	S	Chongqing	1	0	no	no
36	3.28 *	3.63	12.67	9.01	4.97	1032	0.530	S	Guangdong	1	0	no	no
37	3.58	3.85	13.57	9.74	5.40 †	1035	0.566	S	Guangdong	1	0	no	no
38	3.47	4.32	13.73	9.44	5.21	1033	0.546	E	Jiangsu	0	0	yes	yes
39	3.60	3.86	13.40	9.55	5.18	1033	0.553	NE	Heilongjiang	0	0	yes	yes
40	3.55	4.20	13.55	9.35	5.02	1032	0.540	E	Shandong	0	0	yes	no
41	3.40	3.72	13.08	9.36	5.20	1033	0.548	NW	Xinjiang	0	0	yes	no
42	3.40	4.40	13.56	9.16	4.99	1032	0.536	S	Guizhou	0	0	yes	no
43	3.51	4.22	13.83	9.65	5.36	1034	0.560	N	Hebei	0	0	no	yes

^a^ ID, sample identification; TS, total solids; SNF, solids nonfat; FPD, freezing point depression; KNN, k-nearest neighbours. S, South; E, East; NW, Northwest; NE, Northeast and N, Central North. The colours dark-red, light-red and green indicate the worst-to-no anomalies detected for the univariate boundaries, and the colours red and green indicate the abnormal samples marked by the KNN models. * The value exceeding the lower measured boundary. ** The value exceeding the lower variance-adjusted boundary. † Value exceeding the upper measured boundary.

**Table 4:** The geographical prevalence of the suspected samples (flagged by the three criteria in Table 3), the mean ranks of scores of the fraud factors showing significant differences between the four geographical areas ^a^ and the principal component regression (PCR) results.

Parameters		East	Central-North	North-West	North-East	Variable Coefficients ^d^
Percentage (%) of suspected samples in the market survey set (number of suspected/total samples)		38% (3/8)	31% (4/13)	13% (2/15)	0% (0/2)	-
Fraud factors on opportunities and motivations ^b^	1. Available technology for milk adulteration	49	46	71	66	−0.201
	2. Detectability of adulteration	51	56	35	58	−0.056
	3. Accessibility to production activities	53	49	57	63	−0.243
	4. Relationships within the supply chain	47	61	39	36	0.174
	5. Valuable components/attributes	38	62	39	42	0.023
	6. Farmer’s financial pressure imposed by the company	40	61	40	45	0.005
	7. Level of competition	73	41	73	53	0.096
	8. Price difference due to regulatory differences	62	47	67	48	0.125
Fraud factors on Controls ^c^	9. Application of integrity screening of employees in the company	51	46	70	63	−0.172
	10. Strictness of the ethical code of conduct in the company	45	48	70	61	−0.188
	11. Support of a whistle-blowing system in the company	62	47	69	48	0.115
	12. Specificity of the national food policy	70	48	55	49	0.209
	13. Availability of a fraud contingency plan	62	45	65	61	−0.051

^a^ The fraud vulnerability data was retrieved from [11], based on 104 milk production participants (90 farmers and 14 milk processors) in China. ^b^ Higher rank of the opportunities and motivations factors indicate higher vulnerability [11]. ^c^ Higher rank of the control factors indicate more adequate controls and, thus, lower vulnerability [11]. ^d^ The variable coefficients in the regression vector of the principal component regression (PCR) between the mean rank of the scores of the fraud factors and the percentage of the suspected samples for the four geographical areas.

## Supplementary Materials

The following are available online at https://www.mdpi.com/2304-8158/9/6/709/s1: Table S1. The calculations and four levels of adulterants added into the three milk pools (per 100 g). Table S2. The amount of adulterants used for the combined-adulterations for the three milk pools. Table S3. The result of the milk compositions and features of the control samples for the measured dataset. Table S4. The result of the milk compositions and features of the control samples for the variance-adjusted dataset.
