# Supplementary material for: Prevalence of Milk Fraud in the Chinese Market and its Relationship with Fraud Vulnerabilities in the Chain
Source: Foods. 2020 Jun 1;9(6):709. doi: 10.3390/foods9060709 (PMC7353633; doi:10.3390/foods9060709)
Supplement: Supplementary file 1 [file foods-09-00709-s001.pdf]

Table 1. The calculations and four levels of adulterants added into the three milk pools (per 100 g).

Adulterant category	Calculation	Adulterant	Level 1 (g)	Level 2 (g)	Level 3 (g)	Level 4 (g)
Protein-rich adulterants	$Weight_{adulterant} = 100\ g \times \left(\frac{Protein_{control} \times a\%}{Protein_{adulterant}}\right)$	WMP	1.36	2.72	4.09	5.45
		SMP	0.99	1.98	2.97	3.95
		WPI	0.38	0.75	1.13	1.51
		SOY	0.39	0.78	1.17	1.56
		PEA	0.43	0.85	1.28	1.71
Nitrogen-based adulterants	$Weight_{adulterant} = 100\ g \times \left(\frac{Protein_{control} \times a\%}{f \times N_{adulterant}}\right)$	URE	0.12	0.24	0.35	0.47
		MLM	0.08	0.16	0.25	0.33
		AC	0.21	0.42	0.63	0.84
		AS	0.26	0.52	0.78	1.04
		DIC	0.08	0.16	0.25	0.33
Carbohydrate-based adulterants	$Weight_{adulterant} = 100\ g \times \left(\frac{TS_{control} \times a\%}{TS_{adulterant}}\right)$	SU	1.30	2.60	3.90	5.20
		GLU	1.30	2.60	3.90	5.20
		FRU	1.30	2.60	3.90	5.20
		LAC	1.30	2.60	3.90	5.20
		MD	1.30	2.60	3.90	5.20
		STA	1.30	2.60	3.90	5.20
		AR	1.53	3.06	4.59	6.12
Preservatives	Not available	CIT	0.05	0.10	0.15	0.20
		CAR	0.05	0.10	0.15	0.20
		BIC	0.05	0.10	0.15	0.20
		FMD	0.05	0.10	0.15	0.20
		PX	0.05	0.10	0.15	0.20
		HYD	0.05	0.10	0.15	0.20
Water	Not available	Water	10.00	20.00	30.00	40.00

a% stands for the four adulteration levels for the protein-rich, nitrogen-based and carbohydrate-based adulterants, it is equal to 10%, 20%, 30% and 40% for the four levels, respectively.

$Protein_{control}$ is the protein content of the control milk samples (3.5% w/w).

$Protein_{adulterant}$ is the protein content of the protein-rich adulterant.

$N_{adulterant}$ is the nitrogen content of the nitrogen-based adulterant.

f is the conversion factor of nitrogen to protein (6.38).

$TS_{control}$ is the total solids content of the control milk samples (13.0% w/w).

$TS_{adulterant}$ is the total solids content of the carbohydrate-based adulterant.

AC: ammonium chloride; AR: arrowroot powder; AS: ammonium sulphate; BIC: sodium bicarbonate; CAR: sodium carbonate; CIT: sodium citrate; DIC: dicyandiamide; FMD: formaldehyde; FRU: fructose; GLU: glucose; HYD: Sodium hydroxide; LAC: lactose; MD: maltodextrin; MLM: melamine; PEA: pea protein isolate; PX: hydrogen peroxide; SMP: skimmed milk powder; SOY: soy protein isolate; STA: starch; SU: sucrose; URE: urea; WMP: whole milk powder; WPI: whey protein isolate.

Table 2. The amount of adulterants used for the combined-adulterations for the three milk pools.

Adulterant category	Calculation	Adulterant	Amount of adutlerant (g)	Pooled milk (g)	Water (g)
Protein-rich adulterants	$Weight_{adulterant} = \frac{\{Protein_{control} \times (1 + a\%) \times (100 + 40)\ g\} - (Protein_{control} \times 100\ g)}{Protein_{adulterant}}$	WMP	13.07	100.00	40.00
		SMP	9.49	100.00	40.00
		WPI	3.61	100.00	40.00
		SOY	3.73	100.00	40.00
		PEA	4.10	100.00	40.00
Nitrogen-based adulterants	$Weight_{adulterant} = \frac{\{Protein_{control} \times (1 + a\%) \times (100 + 40)\ g\} - (Protein_{control} \times 100\ g)}{f \times N_{adulterant}}$	URE	1.13	100.00	40.00
		MLM	0.79	100.00	40.00
		AC	2.01	100.00	40.00
		AS	2.49	100.00	40.00
		DIC	0.79	100.00	40.00
Carbohydrate-based adulterants	$Weight_{adulterant} = \frac{\{TS_{control} \times (1 + a\%) \times (100 + 40)\ g\} - (TS_{control} \times 100\ g)}{TS_{adulterant}}$	SU	12.48	100.00	40.00
		GLU	12.48	100.00	40.00
		FRU	12.48	100.00	40.00
		LAC	12.48	100.00	40.00
		MD	12.48	100.00	40.00
		STA	12.48	100.00	40.00
		AR	14.68	100.00	40.00

a% is equal to 40% for the combined-adulteration.
$Protein_{control}$ is the protein content of the control milk samples (3.5% w/w).
$Protein_{adulterant}$ is the protein content of the protein-rich adulterant.
$N_{adulterant}$ is the nitrogen content of the nitrogen-based adulterant.
f is the conversion factor of nitrogen to protein (6.38).
$TS_{control}$ is the total solids content of the control milk samples (13.0% w/w).
$TS_{adulterant}$ is the total solids content of the carbohydrate-based adulterant.
AC: ammonium chloride; AR: arrowroot powder; AS: ammonium sulphate; DIC: dicyandiamide; FRU: fructose; GLU: glucose; LAC: lactose; MD: maltodextrin; MLM: melamine; PEA: pea protein isolate; SMP: skimmed milk powder; SOY: soy protein isolate; STA: starch; SU: sucrose; URE: urea; WMP: whole milk powder; WPI: whey protein isolate.

Table 3. The result of the milk compositional features of the control samples for the measured dataset.

Sample ID	Protein (% w/w)	Fat (% w/w)	TS (% w/w)	SNF (% w/w)	Lactose (% w/w)	Density (g/L)	FPD (°C)
1	3.61	3.72	13.36	9.71	5.36	1035	0.562
2	3.71	4.43	14.03	9.67	5.21	1033	0.562
3	3.72	4.21	13.98	9.86	5.40	1035	0.570
4	3.74	4.35	13.95	9.66	5.18	1033	0.569
5	3.49	3.88	13.02	9.17	4.96	1032	0.530
6	3.42	3.93	12.86	8.94	4.80	1031	0.516
7	3.55	3.99	13.10	9.13	4.84	1031	0.522
8	3.34	3.60	12.56	8.98	4.91	1031	0.521
9	3.72	4.04	13.75	9.78	5.31	1034	0.576
10	3.33	3.75	12.71	8.99	4.93	1032	0.521
11	3.42	3.97	12.98	9.03	4.88	1031	0.529
12	3.42	3.73	12.82	9.11	4.96	1032	0.538
Pool A	3.69	4.05	13.72	9.73	5.30	1034	0.567
Pool B	3.44	3.75	12.79	9.05	4.88	1031	0.524
Pool C	3.47	3.83	13.04	9.24	5.04	1032	0.544

ID: Identity; FPD: freezing point depression; TS: total solids; SNF: solids non-fat.

Table 4. The result of the milk compositional features of the control samples for the variance-adjusted dataset.

Sample ID	Protein (% w /w)	Fat (% w/w)	TS (% w/w)	SNF (% w/w)	Lactose (% w/w)	Density (g/L)	FPD (°C)
1	3.67	3.48	13.47	10.09	5.66	1038	0.581
2	3.88	4.91	14.81	10.00	5.35	1034	0.581
3	3.89	4.46	14.72	10.39	5.73	1038	0.597
4	3.93	4.74	14.66	9.99	5.29	1034	0.595
5	3.43	3.81	12.80	9.01	4.85	1032	0.517
6	3.29	3.91	12.48	8.55	4.54	1030	0.489
7	3.56	4.02	12.95	8.92	4.62	1030	0.500
8	3.14	3.24	11.88	8.62	4.76	1030	0.499
9	3.90	4.12	14.26	10.23	5.56	1036	0.609
10	3.12	3.54	12.18	8.64	4.79	1031	0.499
11	3.30	3.98	12.72	8.72	4.70	1030	0.515
12	3.30	3.51	12.39	8.88	4.85	1032	0.533
Pool A	3.83	4.14	14.19	10.13	5.54	1036	0.591
Pool B	3.34	3.55	12.34	8.77	4.70	1030	0.505
Pool C	3.39	3.71	12.83	9.14	5.01	1032	0.545

ID: Identity; FPD: freezing point depression; TS: total solids; SNF: solids non-fat.
